# Dysregulated Neuroimmune and Anhedonia-Like Behavioral Response Following Peripheral Immune Challenge in Mice Carrying the Val66Met Brain-Derived Neurotrophic Factor Polymorphism

**DOI:** 10.3390/psychiatryint6030087

**Published:** 2025-07-21

**Authors:** Mustafa N. Mithaiwala, Allison M. Dugan, Miguel A. de la Flor, Ashley Acheson, Sandeep K. Subramanian, Jason C. O’Connor

**Affiliations:** 1 Department of Pharmacology and Center for Biomedical Neuroscience, University of Texas Health Science Center at San Antonio, San Antonio, TX 78229, USA; 2 Department of Psychiatry and Behavioral Science, University of Arkansas for Medical Sciences, Little Rock, AR 72205, USA; 3 Department of Physical Therapy, University of Texas Health Science Center at San Antonio, San Antonio, TX 78229, USA; 4 Geriatric Research, Education and Clinical Center, South Texas Veterans Health System, San Antonio, TX 78229, USA

**Keywords:** Val66Met Polymorphism, BDNF, LPS, Inflammation, Cytokines, Anhedonia

## Abstract

Dysregulated inflammatory processes contribute to depression, and gene–environment interactions may influence an individual’s risk and resilience. Reduced brain-derived neurotrophic factor (BDNF) expression increases susceptibility for developing depressive symptoms, and the Val66Met (rs6265) single-nucleotide polymorphism (SNP) on the BDNF gene is linked to mood disorders. However, whether Val66Met confers increased vulnerability to inflammation-induced depressive tendencies is unknown. Here, we tested the hypothesis that the Val66Met SNP increases vulnerability to inflammation-induced depressive symptoms in a mouse model of lipopolysaccharide (LPS)-induced depression-like behavior. Behavior and neuroinflammation, following a 24 h LPS challenge, were measured in mice expressing the human BDNF Val66Met gene variant or Val66Val littermates (control). The Val66Met genotype did not affect the peripheral inflammatory response, acute neuroinflammation, or the acute sickness behavior response. Val66Met mice exhibited anhedonia-like behavioral responses following LPS challenge, and we found increased mRNA expression of IL-1β and TNFα in the cerebrum compared to controls. The mRNA expression of IL-1β and TNFα in the hippocampus and the nucleus accumbens of Val66Met mice was increased following LPS, and a significant genotype ×LPS interaction was detected for CD68 expression in the nucleus accumbens. In summary, these data suggest that immune activation in Val66Met mice increased susceptibility to anhedonic behavior and dysregulated negative regulation of inflammation.

## 1. Introduction

Depression is the leading cause of disability worldwide [1], and approximately 15–30% of patients experiencing depressive episodes respond inadequately to first-line therapeutics [2]. Inflammation can induce depressive episodes [3], and increased inflammation can predict depressive symptoms [4]. Depressed patients with defective anti-inflammatory immune responses respond inadequately to antidepressant treatment [5], and the blockade of inflammation (tumor necrosis factor antagonist-infliximab) has shown antidepressant efficacy in depressed patients with elevated C-reactive protein (CRP) levels [6]. Meta-analyses indicate that the inflammatory marker CRP is frequently elevated in individuals experiencing depression despite adjustment for epidemiological and other confounding factors. The increased CRP levels are associated with physical (appetite and energy) and cognitive symptoms of depression (lack of interest in activities); however, peripheral inflammation is not associated with the emotional symptoms (hopelessness and feeling like a failure) of depression [7]. Thus, the variability in the severity of depressive symptoms experienced by individuals could be related to interactions between biological risk factors, such as genetics, and other environmental factors, such as inflammation or chronic stress. An understanding of the mechanisms underlying such interactions is needed to advance the tools required for personalized psychiatric and clinical care.

The neurotrophin hypothesis of depression postulates that brain-derived neurotrophic factor (BDNF) is involved in depression, and the implications of reduced/impaired BDNF are well established in human and animal studies [8]. Reduced serum levels of BDNF were observed in patients suffering from major depressive disorder [9], and reduced brain gene transcript and protein levels of BDNF were documented in young suicide victims [10]. Increased DNA methylation of the BDNF gene in peripheral blood cells of major depressive disorder patients suppresses BDNF expression, suggesting that epigenetic changes to this neurotrophic gene may disrupt physiological BDNF-Tropomyosin receptor kinase B (TrkB) signaling [11]. Patients with depressive episodes exhibit reduced BDNF levels in the serum [12] and in the brain [13], while effective treatment with antidepressants increases BDNF levels in the brains of depressed patients [14]. In rodent models, chronic unpredictable stress induces depressive-like behaviors concomitant with reduced levels of mature BDNF and its receptor TrkB in the neocortex and the hippocampus [15]. However, the efficacy of an antidepressant, like fluoxetine, to rescue depressive behaviors and increase BDNF mRNA is dependent on the environmental factors in which treatment is presented, and outcomes vary between stressful and stress-free conditions [16]. Furthermore, previous investigations studying BDNF heterozygous mice suggest that exposure to stress induces the development of depressive-like behaviors [17,18], and that a genetic reduction in BDNF is associated with an exaggerated neuroimmune response to peripheral immune challenge [19]. While the relationship between the BDNF and neuroinflammation may be bidirectional in nature [20], the cellular and molecular mechanisms altered by such a gene (BDNF) and environment (immune challenge) interaction remain widely unknown.

A common single-nucleotide polymorphism (SNP), rs6265, involving a valine for methionine substitution at amino acid position 66 (Val66Met) on the brain-derived neurotrophic factor (BDNF) gene is associated with the development of dysthymic mood disorders [21] and the onset of cognitive [22] and memory deficits [23]. The Val66Met SNP impairs neuronal processing and reduces activity-dependent release of mature BDNF [23–25]. The BDNF Val66Met genotype is also linked to a negative modulation of early life experiences, increasing the risk of developing depression in adulthood [26,27]. As such, the Val66Met SNP may be a vulnerability factor for stress-induced [28] or inflammation-induced depression [29]. Patients carrying the Val66Met allele were more likely to develop depressive symptoms after treatment with interferon-α [30], and the Met allele predicted inflammation-associated depressive symptoms in women with breast cancer [31]. In preclinical models, mice expressing the human BDNF Val66Met SNP under the endogenous BDNF promoter are more susceptible to anxiogenic stimuli, stress-induced depressive-like behaviors, and less responsive to antidepressant treatment [25,32]. These data suggest that the Val66Met SNP may sensitize the development of depressed mood associated with an aberrant inflammatory tone.

To directly investigate the interaction between the Val66Met SNP and neuroinflammation, we utilized mice expressing the human BDNF gene with or without the Val66Met SNP in the well-validated endotoxin (LPS)-induced depressive-like behavioral paradigm. In this study, we tested the hypothesis that the presence of the Val66Met SNP increases vulnerability to inflammation-induced depressive-like behaviors. Peripheral LPS challenge-induced changes were investigated using validated behavioral assays, and peripheral inflammatory responses were analyzed by the measurement of changes in circulating cytokine levels and inflammatory factors in the whole brain, hippocampus, and nucleus accumbens. In this study, we aimed to provide insight into how Val66Met confers vulnerability to inflammation-induced depressive symptoms.

## 2. Methods and Materials

### 2.1. Animals

All animal care and use procedures were carried out in accordance with the Guide for the Care and Use of Laboratory Animals, 8th edition (NRC) and approved by the Institutional Animal Care and Use Committees at the Audie L. Murphy VA Hospital and UT Health San Antonio. Male 10–16-week-old heterozygous BDNF Val66Met mice and wild-type Val66Val littermate controls on a C57BL/6J background were utilized in all experiments. The breeding colony was maintained by crossing heterozygous Val66Met male mice with wild-type Val66Val females. Pilot experiments identified differences in the baseline behavioral phenotype of female mice, which precluded the combination of male and female mice in the same groups; however, funding support was insufficient to include a full analysis of female mice. Thus, all experiments in this study were conducted in young-adult male mice. Mice were group-housed in standard shoebox cages within a ventilated caging system. Mice were allowed *ad libitum* access to food and water and maintained under a reverse 12:12 h light/dark cycle with lights out at 11:00 am. All animals used in this study were weighed and handled daily for 5 min per mouse beginning three days prior to injections and subsequent behavioral testing. This handling helps to minimize stress and habituate mice to the experimenter. Animals were moved from the vivarium to the testing room 1 h before testing began to acclimatize to the environment where testing was conducted. Cages were covered with an opaque curtain during transport to minimize disruptions in circadian rhythms. All behavioral testing was conducted during the first four hours of the dark phase under low light conditions (<100 lux) to minimize stress associated with light, and animals were returned to home cages and kept separated from animals undergoing testing.

### 2.2. Genotyping

DNA from experimental mice was used for genotyping using the following protocol: 8.5 μL JumpStart Taq (Sigma-Aldrich, St. Louis, MO, USA), 0.85 μL primers (12 μM), 4.4 μL PCR-grade water, and 2 μL DNA. The following primer sequences were used (Integrated DNA Technologies, Coralville, IA, USA): common forward TCA TAC TTC GGT TGC ATG AAG G, Val reverse ATC CAG CAG CTC TTC GAT GAC G, and Met reverse ATA AAT CCA CTA GTG GTG GTG G. Upon receipt of the primers, they were reconstituted using PCR-grade water to 100 μM, and aliquots were stored at −20 °C. Primers remained stable for about 4 months. PCR was performed in a thermocycler (C1000 Bio-Rad, Hercules, CA, USA): step (1) 94 °C, 3 min, step (2) 94 °C 1 min, step (3) 55 °C, 1 min, step (4) 72 °C, 1 min, step (5) repeat steps 2–4 39x×, step (5) 72 °C, 5 min, step (6) hold at 10 °C. Loading dye was added to samples, which were then run at 75 V for 30 min through a 2.5% agarose gel; DNA bands were visualized using SyberSafe reagent (ThermoFisher, Waltham, MA, USA) and UV light.

### 2.3. Experimental Timeline and Treatments

**Figure 1.**
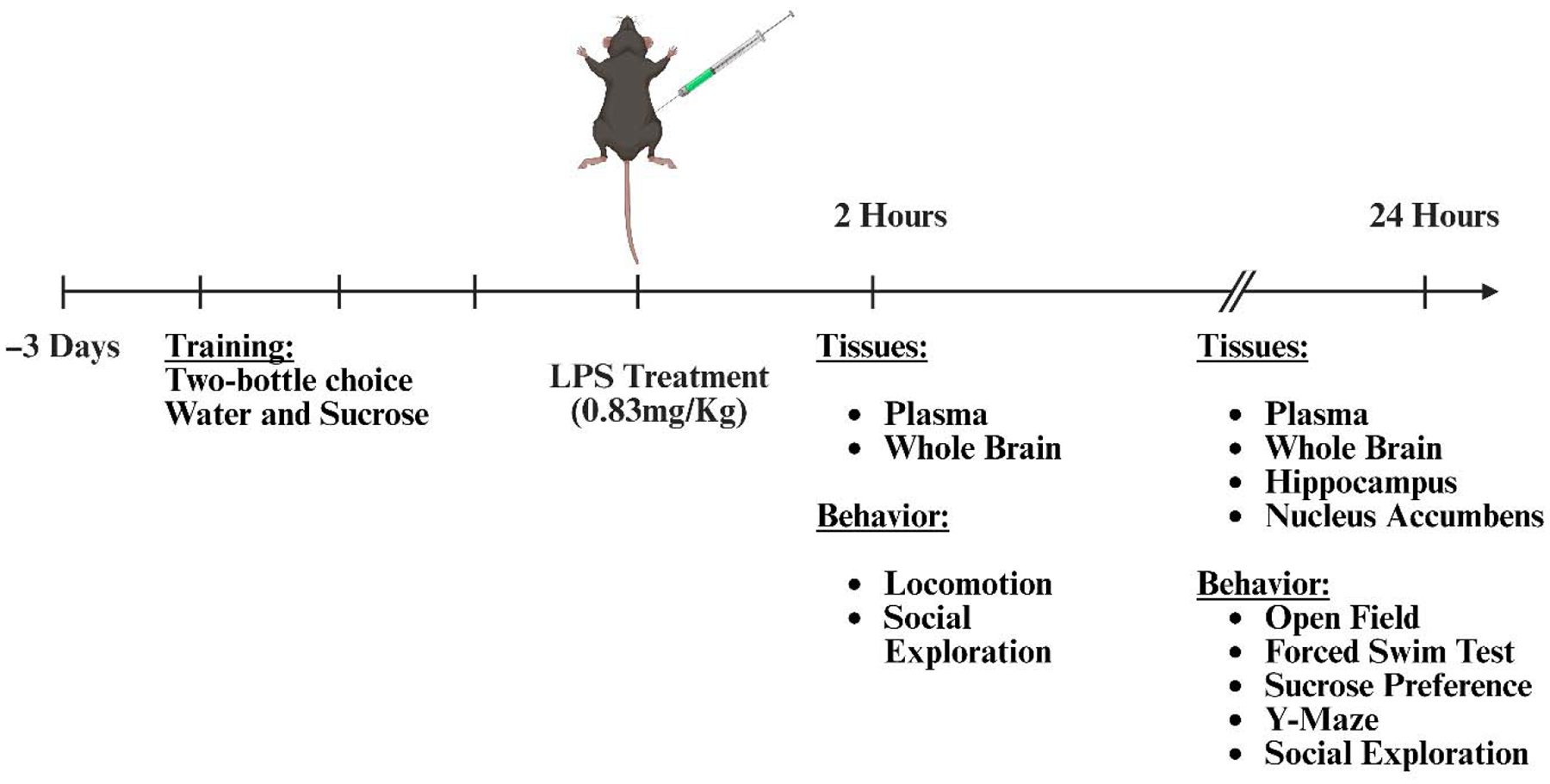
Experimental design and timeline. Three days prior to LPS injection, mice were trained for sucrose preference. Two hours after LPS, locomotion and social exploratory behavior were measured, and plasma and brains were collected. Twelve hours after LPS, the whole brain was collected. Then, 24 h following LPS, locomotor activity, social exploration, depressive-like behavior, and working memory were measured, and plasma, brain, hippocampus, and nucleus accumbens were collected.

Young Val66Val and Val66Met male mice (10–16 weeks old) on a C57BL/6J background were injected intraperitoneally (ip.) with 0.83 mg/kg lipopolysaccharide (LPS, *E. coli* 0127: B8, Sigma-Aldrich, Germany) or vehicle (0.9% sterile saline, Bound Tree Medical, Dublin, OH, USA), as illustrated in Figure 1. Then, 2 or 24 h after treatment, separate groups of mice were subjected to behavioral testing as described below. For molecular and cellular analyses, mice were euthanized at the indicated times after treatment, blood samples were collected from the inferior vena cava, and the mice were perfused transcardially with ice-cold heparinized saline. The whole brains were removed, and tissue was prepared for subsequent analysis.

### 2.4. Behavior Testing

#### Sucrose Preference:

Anhedonia-like behavior was measured as previously described [33]. Briefly, mice (*n* = 10 mice/group) were trained using a two-bottle free-choice paradigm (1% sucrose or water) for three days prior to treatment. Sucrose preference was calculated as the 24 h change in weight of sucrose/(weight of sucrose + water) * 100 following LPS challenge.

#### Open Field:

To evaluate locomotor activity and anxiety-like behavior, the open field test was used as previously described [33]. Briefly, mice (*n* = 5–7 mice/group) were placed in an open field arena for 5 min, and their locomotive activity was video recorded. Videos were analyzed using Ethovision 8.5 software (Noldus Leesburg, VA, USA).

#### Forced Swim Test:

Behavioral despair-like behavior was measured as previously described [34]. Briefly, mice (*n* = 6–7 mice/group) were placed in a 4 L Nalgene beaker of water (25 °C ± 1) for 5 min, and their activity was video recorded from above. A trained observer blinded to experimental treatments then scored the videos by measuring the time the mouse spent immobile.

#### Social Exploration:

To measure acute sickness-like behavior, the social interaction of a novel conspecific was measured as previously described [35] with minor modifications. Briefly, after habituation to an open field arena with an empty inverted mesh cup in the corner for 15 min, a novel same-sex conspecific was placed under the cup, and the activity of the test mouse was video recorded for 10 min (*n* = 9–11 mice/group). Videos were analyzed using Ethovision 8.5.

#### Y-Maze:

Short-term working memory was assessed using a two-trial Y-maze task [36]. Distinct visual intra-maze cues [37] were posted at the end of each arm during a 15 min exposure trial in which one of the arms was blocked. After 1 h, the mouse was reintroduced to the same maze with free access to all three arms and video-recorded for 5 min. Videos were analyzed using Ethovision 8.5. The discrimination ratio was calculated as (time spent in novel arm)/(time spent in novel + familiar arms) (*n* = 8–10 mice/group).

### 2.5. Tissue Preparation and RT-qPCR

Saline perfused brain tissue was flash frozen in liquid nitrogen and stored at −80 °C until homogenization in RNA lysis buffer from the PureLink RNA mini kit (ThermoFisher) using a BeadRuptor (Omni International, Kennesaw, GA, USA). Samples were then centrifuged at 4 °C for 10 min at 1500× *g* to pellet the beads and insoluble tissue pieces. The supernatant was removed for RNA isolation according to the manufacturer’s instructions. Brain hemispheres were micro-dissected to collect the hippocampus and nucleus accumbens according to stereotaxic coordinates in the Franklin and Paxinos mouse brain atlas [38] and then processed as described above.

RNA concentrations were determined using a spectrophotometer (NanoVue Plus, GE Healthcare, Chicago, IL, USA), and then equal concentrations of RNA were used for reverse transcription to generate cDNA using the High-Capacity cDNA reverse transcription kit (Applied Biosystems, Waltham, MA, USA). cDNA was used in RT-qPCR using Taqman primers and probes (ThermoFisher; Gapdh Mm99999915, Il1b Mm00434228_m1, Tnfa Mm00443258, Il6 Mm00446190, Aif1 Mm00479862, Cd68 Mm03047343_m1, Nos2 Mm00440502, IL4Ra Mm01275139_m1, Il1Rn Mm00446186_m1). RT-qPCR was performed using the Bio-Rad CFX 384 system. Data are expressed as fold change relative to Val66Val saline controls using the ΔΔCt method [39].

### 2.6. Enzyme-Linked Immunosorbent Assay

Plasma concentrations of the following pro-inflammatory cytokines, interleukin-1β (IL-1β), interleukin-6 (IL-6), and tumor necrosis factor-α (TNFα) were determined using ELISA kits (Invitrogen, Waltham, MA, USA) according to the manufacturer's instructions.

### 2.7. Statistical Analysis

Data were checked to ensure that normality of distribution assumptions were met using the Shapiro–Wilk test. All data were analyzed via GraphPad Prism10.4.1 (San Diego, CA, USA) using a two-way ANOVA with the genotype and LPS challenge as the two factors when assumptions of normality were met. If there was a significant interaction between the factors, Tukey’s multiple comparison post hoc test was used to determine significant differences between the means of the different treatment groups. Non-normally distributed data were analyzed using the non-parametric Kruskal–Wallis’s test, and for post hoc testing, the corrected Dunn’s multiple comparison test was used to determine significant differences between the means of the different genotypes and treatment groups. Data were considered significant if *p* < 0.05. Spurious data were identified using Chauvenet’s outlier criteria [40], and a total of 5 animals were excluded from data analysis.

## 3. Results

### 3.1. BDNF Val66Met Expression Results in Susceptibility to the Anhedonia-Like Behavioral Effects of Immune Challenge

**Figure 2.**
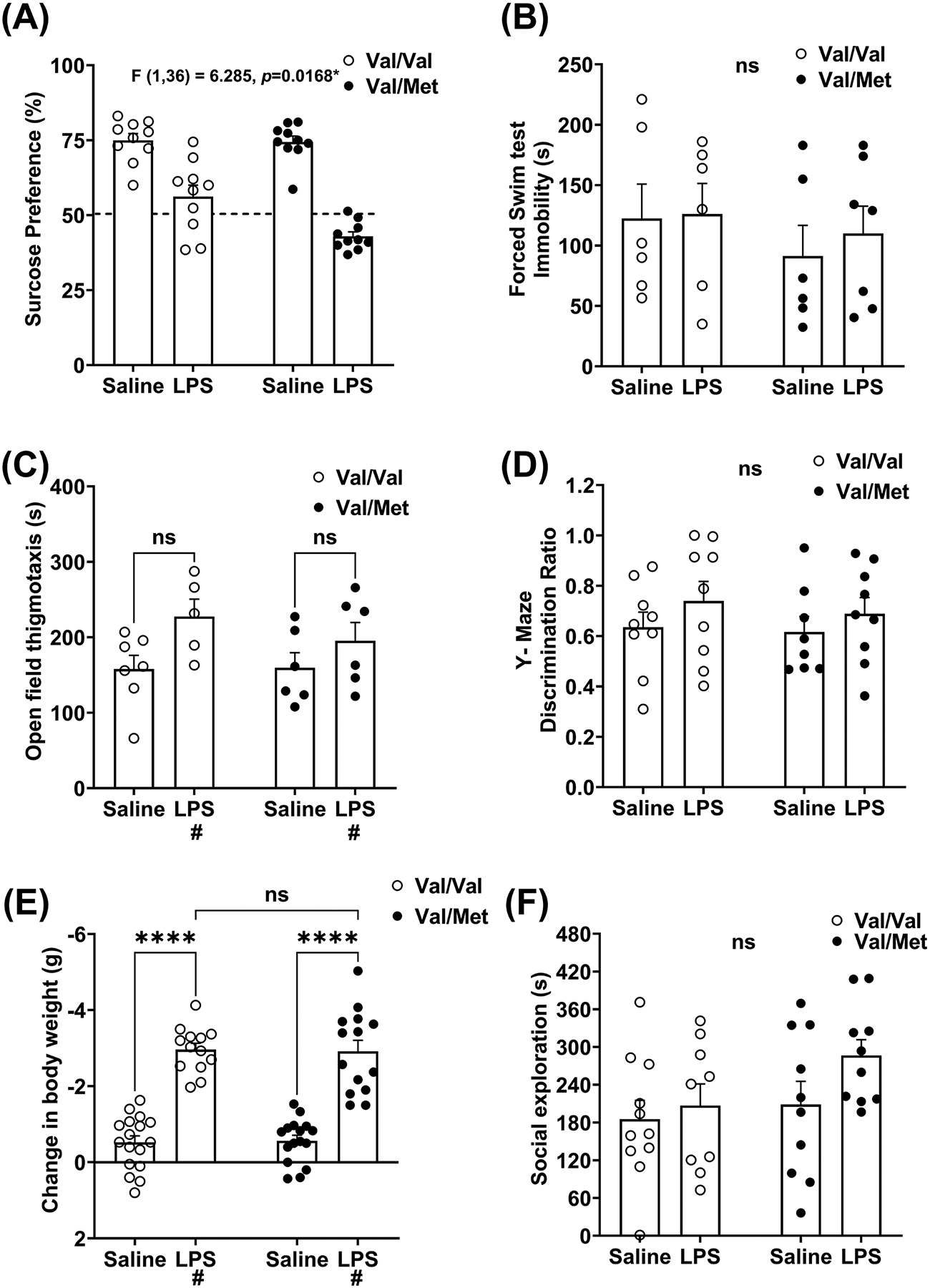
BDNF Val66Met expression results in susceptibility to the anhedonia-like behavioral effects of immune challenge. 24 h after intraperitoneal saline or LPS administration, the following metrics were measured, and data were analyzed using a two-way ANOVA with genotype and LPS as independent factors for normally distributed data, and a non-parametric Kruskal–Wallis’s test was used to analyze non-normally distributed data. (**A**) Sucrose preference; dotted line is at the 50% mark, which indicates no preference (*n* = 10 mice/group); two-way ANOVA interaction: genotype × LPS: F (1,36) = 6.285, *p* = 0.0168, η^2^p = 0.149. (**B**) Forced swim test (Kruskal–Wallis’s test, *p* = 0.6397*, n* = 6–7 mice/group). (**C**) Open field test (*n* = 5–7 mice/group); main effect of LPS: F (1,20) = 6.088, *p* = 0.0228, η^2^p = 0.407. (**D**) Discrimination ratio in Y-maze working memory task (*n* = 8–10 mice/group). (**E**) Change in body weight (*n* = 13–17 mice/group); main effect of LPS: F (1,56) = 146.2, *p* < 0.0001, η^2^p = 0.723. (**F**) Social exploratory behavior (*n* = 9–11 mice/group). Error bars represent +/− standard error of means (SEM). Post hoc testing with Tukey’s multiple comparison test to compare differences between group means if main effects of genotype or LPS were detected and for non-normally distributed data post hoc testing with corrected Dunn’s multiple comparison test was used to compare differences between group means; # represents main effect of LPS; ns—not significant; * *p* < 0.05, **** *p* < 0.0001.

Following an intraperitoneal LPS challenge, Val66Met mice did not exhibit a preference for sucrose solution over water, and their sucrose preference was significantly lower than saline-treated same-genotype controls or Val66Val mice challenged with LPS (Genotype × LPS interaction: F (1,36) = 6.285; *p* = 0.0168, η^2^p = 0.149; Figure 2A). The duration of immobility (non-normal distribution) in the forced swim test (FST) was not different between saline-treated controls, and LPS challenge did not result in differences in the FST between Val66Val and Val66Met mice (Kruskal–Wallis’s test, *p* = 0.6397; Figure 2B). The effect of immune activation (LPS: F (1,20) = 6.088, *p* = 0.0228, η^2^p = 0.407) on thigmotaxic (wall-hugging) behavior persisted in both genotypes in the open field test (Figure 2C), and group means were not different from each other (Tukey’s multiple comparison testing). There were no significant differences in locomotor activity in the open field test 24 h after LPS challenge. In the trial phase of the Y-maze, there were no baseline differences in performance related to the genotype, and LPS challenge did not affect the ability to discriminate between the novel (blocked) versus the familiar (unblocked) (Figure 2D).

Two hours after LPS, mice of both genotypes exhibited sickness-like behavior (See Supplementary material), with a main effect of LPS dampening locomotion (LPS: F (1,20) = 33.81, *p* < 0.0001, η^2^p = 0.628) and social exploration (Kruskal–Wallis’s test *H*(3) = 10.24, *p* = 0.0166; Supplemental Figure S1). Furthermore, 24 h after immune challenge (LPS: F (1,56) = 146.2, *p* < 0.0001, η^2^p = 0.723), both genotypes lost similar amounts of body weight (Figure 2E), and post hoc testing revealed significant differences between saline- and LPS-treated Val66Val mice (*p* < 0.0001) and Val66Met mice (*p* < 0.0001). Moreover, 24 h after immune challenge, no significant changes in locomotor activity or social exploration were observed (Figure 2F). Finally, at 2 and 24 h post-LPS challenge, no significant interactions between treatment and genotype were detected in plasma concentrations of the pro-inflammatory cytokines IL-1β, IL-6, or TNFα (Table 1).

### 3.2. Immune Challenge Induces Dysregulated Whole-Brain Pro-Inflammatory Cytokine Expression in BDNF Val66Met Mice

**Figure 3.**
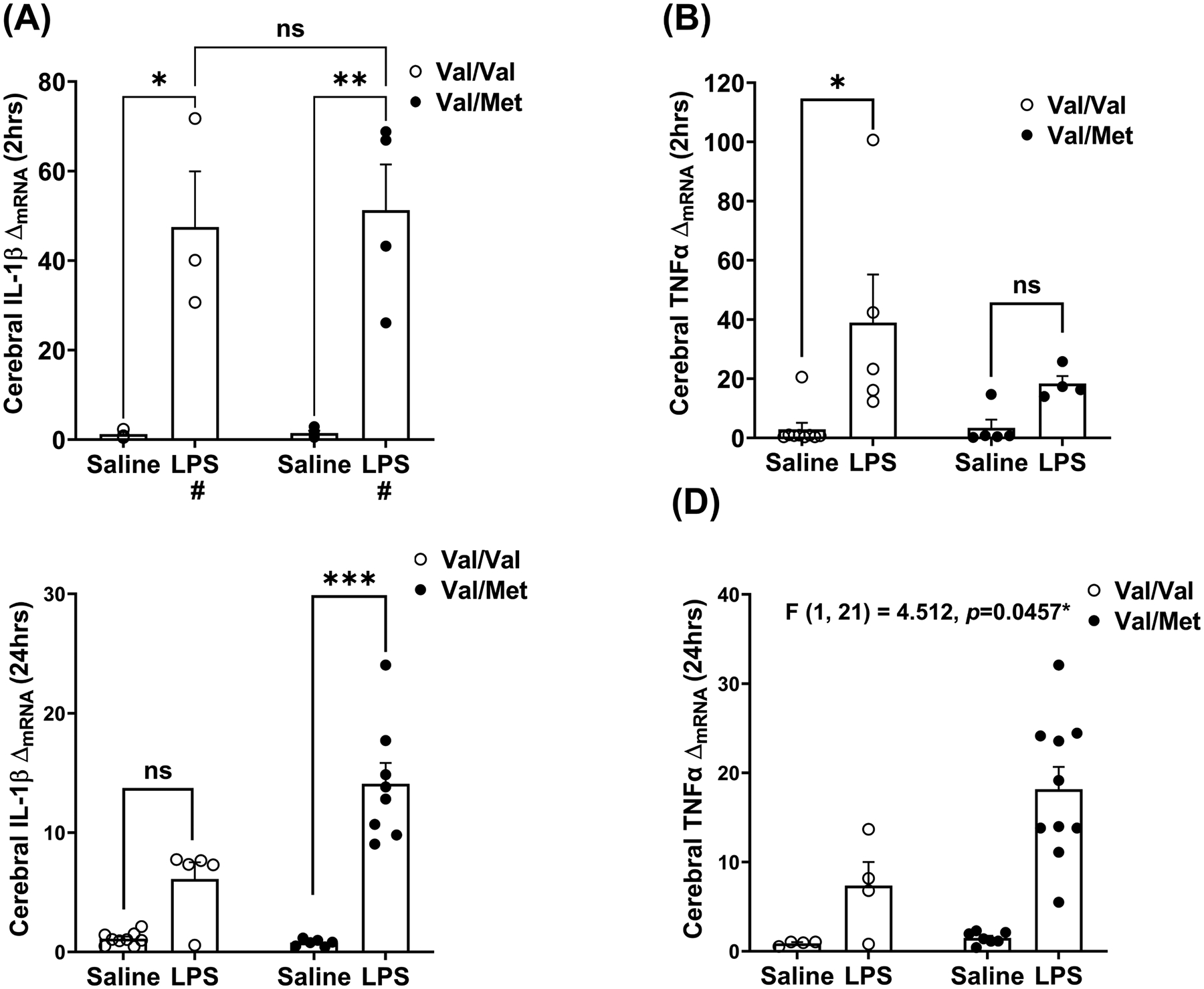
Immune challenge induces dysregulated whole-brain pro-inflammatory cytokine expression in BDNF Val66Met Mice. After 2 or 24 h of saline or LPS administration, the following PCR targets were measured from whole brain, and data were analyzed using a two-way ANOVA with genotype and LPS as the independent factors for normally distributed data, and a non-parametric Kruskal–Wallis’s test was used to analyze non-normally distributed data. The mRNA expression of (**A**) of Interleukin-1β (IL-1β), main effects of LPS: F (1,10) = 36.28, *p* = 0.0001, η^2^p = 0.784; (**B**) tumor necrosis factor-alpha (TNFα); Kruskal–Wallis’s test *H* (3) = 12.63, *p* = 0.0055, η^2^ = 0.574 at 2 h following intraperitoneal saline or LPS administration. The mRNA expression of (**C**) IL-1β, Kruskal–Wallis’s test *H* (3) = 19.85, *p* = 0.0002, η^2^ = 0.735; (**D**) TNFα, genotype ×LPS interaction: F (1,21) = 4.512, *p* = 0.0457, η^2^p = 0.177, at 24 h following intraperitoneal saline or LPS administration. *n* = 3–9 mice/group/timepoint. Error bars represent +/− standard error of means (SEM). Post hoc testing via Tukey’s multiple comparison test was conducted to compare the differences between group means if the main effects of genotype or LPS were detected, and for non-normally distributed data, post hoc testing via corrected Dunn’s multiple comparison test was used to compare the differences between group means; # represents main effect of LPS; ns—not significant; * *p* < 0.05, ** *p* < 0.01, *** *p* < 0.001.

At 2 or 24 h after peripheral LPS challenge, the brain cerebral hemispheres were collected to analyze mRNA expression using real-time RT-PCR (Figure 3). At 2 h, LPS-induced immune challenge increased the expression of the pro-inflammatory cytokines IL-1β (LPS: F (1,10) = 36.28; *p* = 0.0001, η^2^p = 0.784) and TNFα (Kruskal–Wallis’s test *H* (3) = 12.63, *p* = 0.0055, η^2^ = 0.574) (Figure 3A,B). Tukey’s multiple comparison post hoc testing found that LPS-treated Val66Val (*p* = 0.0146) and Val66Met (*p* = 0.0035) mice were different from saline-treated controls on the mRNA expression of IL-1β. The mRNA expression of TNFα was increased in LPS-challenged Val66Val mice (Dunn’s multiple comparison test, *p* = 0.0372) compared to saline controls, but TNFα was not different in the brain tissue of saline- and LPS-treated Val66Met mice.

At 24 h after immune challenge when the LPS-induced sickness behavior is resolved, (Supplemental Figure S1), we detected a significantly higher expression of the pro-inflammatory cytokines IL-1β (Kruskal–Wallis’s test *H* (3) = 19.85, *p* = 0.0002, η^2^ = 0.735) and TNFα (Genotype × LPS: F (1,21) = 4.512, *p* = 0.0457, η^2^p = 0.177) in the brain tissue of LPS-challenged Val66Met mice in comparison to saline-treated controls and Val66Val mice (Figure 2C,D). At 24 h, for changes in mRNA expression of IL-1β, Dunn’s multiple comparison test found significant differences between saline- and LPS-treated Val66Met mice (*p* = 0.0004). There were no significant interactions in the expression of several other pro- and anti-inflammatory factors (Supplemental Table S1).

### 3.3. BDNF Val66Met Mutation Differentially Affects Brain Regions in Response to LPS

**Figure 4.**
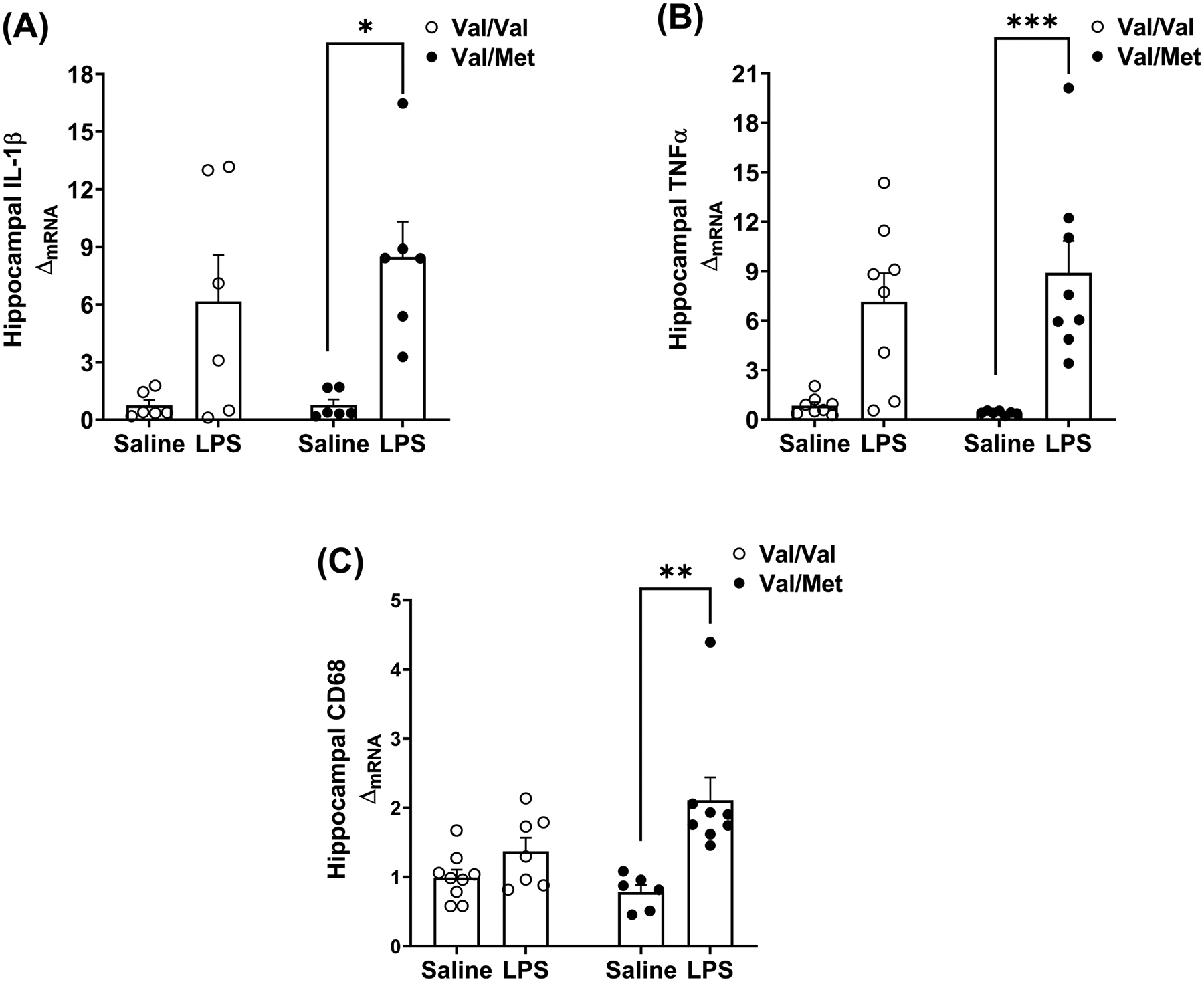
Measurement of pro-inflammatory factors in the hippocampus of Val66Val and Val66Met mice in response to peripheral immune activation. After 24 h, whole brains were micro-dissected, PCR was performed to determine relative gene expression in the hippocampus, and data were analyzed with the non-parametric Kruskal–Wallis’s test. The mRNA expression of (**A**) IL-1β, Kruskal–Wallis’s test *H* (3) = 12.63, *p* = 0.0055, η^2^ = 0.486; (**B**) TNFα, Kruskal–Wallis’s test *H* (3) = 20.55, *p* = 0.0001, η^2^ = 0.735; (**C**) CD68, Kruskal–Wallis test *H* (3) = 16.22, *p* = 0.0010, η^2^ = 0.559*. n* = 4–8 mice/group; post hoc testing with corrected Dunn’s multiple comparison test was used to compare differences between group means; * *p* < 0.05, ** *p* < 0.01, *** *p* < 0.001.

**Figure 5.**
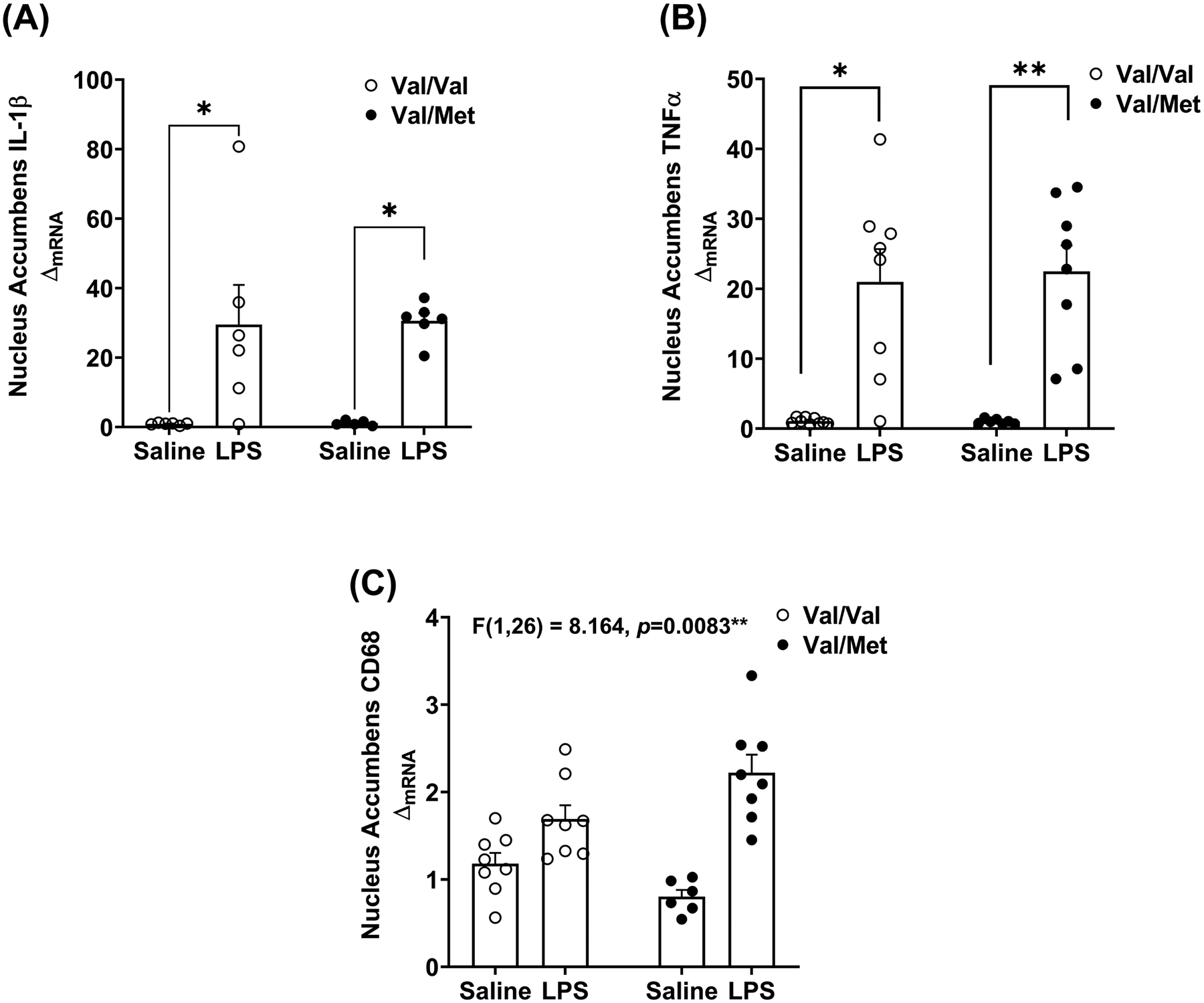
Measurement of pro-inflammatory factors in the nucleus accumbens of Val66Val and Val66Met mice in response to peripheral immune activation. After 24 h, whole brains were micro-dissected and PCR was performed to determine relative gene expression in the nucleus accumbens, and data were analyzed using a two-way ANOVA with genotype and LPS as independent factors for normally distributed data, and a non-parametric Kruskal–Wallis’s test was used to analyze non-normally distributed data. The mRNA expression of (**A**) IL-1β, Kruskal–Wallis’s test *H* (3) = 15.53, *p* = 0.0014, η^2^ = 0.597; (**B**) TNFα, Kruskal–Wallis’s test *H* (3) = 20.98, *p* = 0.0001, η^2^ = 0.677). (**C**) CD68, genotype × LPS interaction: F (1,26) = 8.164, *p* = 0.0083, η^2^p = 0.239*. n* = 4–8 mice/group; post hoc testing with Tukey’s multiple comparison test to compare differences between group means if main effects of the genotype or LPS were detected, and for non-normally distributed data, post hoc testing with corrected Dunn’s multiple comparison test was used to compare differences between group means; * p < 0.05, ** *p* < 0.01.

Steady-state mRNA expression of inflammatory cytokines and the lysosomal/phagocytic marker CD68 was measured in the hippocampus and the nucleus accumbens (Figures 4 and 5). We investigated these brain regions as they are involved in the regulation of behaviors affected in depression [41–44]. We found increased mRNA expression of IL-1β (Kruskal–Wallis’s test *H* (3) = 12.63, *p* = 0.0055, η^2^ = 0.486) and TNFα (Kruskal–Wallis’s test *H* (3) = 20.55, *p* = 0.0001, η^2^ = 0.735) in the hippocampus of Val66Met mice (Figure 4A,B). Similarly, in the nucleus accumbens of Val66Met mice, (Figure 5A,B) LPS challenge influenced the mRNA expression of IL-1β (Kruskal–Wallis’s test *H* (3) = 15.53, *p* = 0.0014, η^2^ = 0.597) and TNFα (Kruskal–Wallis’s test *H* (3) = 20.98, *p* = 0.0001, η^2^ = 0.677). Exploratory post hoc testing found no differences in the expression of these gene transcripts between LPS-treated Val66Val and Val66Met mice.

In the hippocampus, mRNA expression of CD68 (Figure 4C) was higher in LPS-treated Val66Met mice (Kruskal–Wallis’s test *H* (3) = 16.22, *p* = 0.0010, η^2^ = 0.559) but not in saline or LPS Val66Val mice (Figure 3C). In the nucleus accumbens, we found an interaction between the genotype and LPS challenge on the mRNA expression of CD68 (Genotype × LPS F (1,26) = 8.164, *p* = 0.0083, η^2^p = 0.239) (Figure 5C).

## 4. Discussion

In this study, we used the mouse model of LPS-induced neuroinflammation to test the hypothesis that the expression of the Val66Met BDNF SNP increases vulnerability to inflammation-induced depressive-like behaviors. The data suggests that mice expressing the human BDNF gene with Val66Met SNP exhibited pronounced anhedonia-like behavior following immune challenge. Thigmotactic behavior was higher following LPS challenge in both Val66Val and Val66Met genotypes, but LPS challenge did not affect short-term working memory or sociability in either genotype. The cerebral mRNA expression of inflammatory cytokines (IL-1β, TNFα) was higher in Val66Met mice compared to Val66Val mice at 24hrs after LPS challenge.. Further, LPS challenge increased the expression of IL-1β and TNFα in the hippocampus and nucleus accumbens. In these same brain regions, the mRNA expression of CD68 was significantly higher in LPS-challenged Val66Met mice compared to saline-treated genotype controls. Collectively, these results suggest that following an immune challenge, Val66Met mice have higher susceptibility for the development of anhedonia-like behavior and have increased cerebral neuroinflammation.

The role of BDNF in the etiology of depression remains widely debated [45,46]; however, evidence suggests BDNF signaling is involved in the effectiveness of antidepressant drugs [14,32,47]. The Val66Met SNP may increase sensitivity to stress-induced depression [47,48]; meta-analyses have found that Val66Met polymorphism in the BDNF gene moderates the relationship between stress and depression [28]. Genetic factors that interact with external stressors, including stress and inflammation, could be involved in increasing the risk for mood dysregulation [19,20]. Inflammation represents an important risk factor, but little is known regarding genetic contributions that modulate the risk for developing depressive behaviors in response to inflammation. The BDNF Val66Met polymorphism [47,49] and LPS-induced immune activation [33,50] have been independently linked to the development of depressive and anxiety-like behaviors. To date, two clinical studies have linked BDNF Val66Met polymorphism with inflammation-associated depressive symptoms [29,31]. However, whether the Val66Met SNP directly interacts with neuroinflammatory processes to potentiate the depressive-like behavioral effects of inflammation has not been investigated. Here, we found that LPS challenge in Val66Met mice led to a decrease in sucrose preference, suggesting anhedonia-like response independent of sickness-like behavior. LPS-induced neuroinflammation has been shown to increase dopamine synthesis and release in the nucleus accumbens [51], which suggests that these dopamine deficits specifically impair motivational drive required for reward-seeking behavior without interference from malaise or fatigue caused by LPS; instead, they are related to the specific effect of increased pro-inflammatory cytokine production following immune challenge [3,52–54]. Further, we did not find reductions in motivations to complete other behavioral tests like FST, Y-maze, or social exploration, suggesting that at 24 h after LPS challenge, acute sickness behavior was resolved and did not interfere with or influence behavioral testing, which is consistent with our line of inquiry [55]. However, the increased thigmotactic behavior induced by LPS was not affected/influenced by the Val66Met polymorphism. We did not find differences related to Val66Met genotype and LPS challenge on short-term working memory or in social exploratory behavior. Interestingly, our prior study using BDNF heterozygous mice revealed a similar depressive-like phenotypic response to LPS characterized by a reduction in sucrose preference without concomitant neurovegetative-like behavioral potentiation [19]. These data begin to suggest that, perhaps, BDNF activity plays an important role in regulating anhedonia-like behavior during inflammatory conditions.

Recent computational analysis suggests a bi-directional relationship between systemic inflammatory regulators and the development of psychiatric disorders [56]. Peripheral inflammatory signals are communicated to the central nervous system by various routes [55,57,58], including the neural (Vagus nerve)-dependent route and the humoral route via secreted cytokines acting at the blood–brain barrier and across it [51]. In response, infiltrating and brain resident immune cells trigger/orchestrate a neuroinflammatory response characterized by increased production of inflammatory cytokines [59–62]. Using the well-validated LPS-induced immune activation model, we found increased cerebral mRNA expression of IL-1β and TNFα in both Val66Val and Val66Met mice, but the magnitude was significantly higher in the brain tissue of Val66Met mice. This indicates heightened sensitivity in limbic and mesolimbic brain tissue to immune activation in mice carrying the Val66Met polymorphism.

In the hippocampus and in the nucleus accumbens, mRNA expression of IL-1β and TNFα was higher following LPS-induced immune activation in the Val66Met genotype. It is plausible that infiltrating monocytes and macrophages could contribute to differential neuroinflammatory response in the brain, like previously published reports [63–65]; however, additional studies are necessary to confirm this speculation in the context of the Val66Met polymorphism. In the nucleus accumbens, the mRNA expression of CD68 was modulated by both immune activation and the Val66Met polymorphism. CD68 is a known marker of microglia and macrophages during inflammatory states and is critically involved in lysosomal/phagocytic activity of these cells [66–68]. Increased CD68 expression has been linked to stress and inflammation-induced depressive behaviors [69–71]. One study found mice expressing the Val66Met polymorphism to have higher CD68 on myocardial macrophages [72]. To our knowledge, this is the first study to investigate CD68 mRNA expression within the context of neuroinflammation and the BDNF Val66Met polymorphism—we found that CD68 transcripts were modulated by Val66Met polymorphism and LPS challenge, suggesting region-specific neuroinflammatory sensitivity.. We focused and limited our investigations of inflammatory response in the nucleus accumbens and the hippocampus, as several reports have found links between the BDNF Val66Met polymorphism, differentially affecting these brain regions to modulate anxiety-like and depressive-like behaviors [25,47,48,73,74].

Neuroinflammation triggered by injecting LPS directly into the nucleus accumbens of mice is related to growth factors (progranulin) and the NF-κB signaling pathway, which are closely associated with innate immune responses and produce a similar behavioral and neuroinflammatory profile as reported in this study [75,76], and this study provides further evidence for a role of the BDNF Val66Met polymorphism in this context. Within the nucleus accumbens, LPS-triggered neuroinflammation and development of depressive-like behaviors are linked to activity at the dopamine D3 receptor, which is directly associated with motivational and reward-related behaviors like anhedonia, similar to the observations documented here in LPS-treated BDNF Val66Met mice [77]. In contrast, in the hippocampus, we observed increased cytokine transcripts and CD68 only in Val66Met mice injected with LPS, indicating the potential sensitivity of the hippocampus for gene × environment interactions in carriers of the Val66Met SNP. Importantly, the hippocampus is a critical brain region for learning and memory, and while it has been implicated in both anxiety and depressive disorders, our results suggest that anhedonia-like behavior and neuroinflammatory responses are closely related to the interaction between BDNF Val66Met polymorphism and LPS challenge in the nucleus accumbens. Early studies using these Val66Met mice reported extensively on their sensitivity to stress-based challenges or fear-based paradigms. To our knowledge, this study is the first to report, albeit with a limited focus, the functional neuroinflammatory and behavioral consequences of the Val66Met SNP in the LPS-induced depressive-like behavioral paradigm. This study did not include behavioral testing targeting the hippocampus, such as the Barnes maze or learned helplessness, which would help reveal changes in spatial learning and depressive symptoms, respectively [78]. However, it is critical to note that the BDNF Val66Met polymorphism also affects the effectiveness of antidepressants [79,80], especially in the hippocampus, which appears highly sensitive to the Val66Met polymorphism following stress exposure [81–83]. However, the present results are consistent with our previous data reporting a more profound anhedonia-like response to LPS in BDNF +/− mice [19], which suggests potential specificity in the response that involved the nucleus accumbens. Future studies are required to dissect stress v/s immune challenge associations with depressive-like behaviors and relevant changes in inflammatory markers.

Inflammation is increased by myriad factors, and in experimental settings, the activation of the peripheral immune response leads to depressive symptoms in both humans and rodents [51,55,84,85]. The association between inflammatory cytokines and depression has been well described, with alterations in serum and plasma levels of IL-1β, IL-6, IFNγ, and C-reactive protein (CRP) as common indicators of perturbed inflammatory tone and associated depressive symptoms [53,86]. In response to peripheral inflammatory mediators, the resident immune cells of the CNS, microglia, in turn, increase the upregulation and secretion of pro-inflammatory cytokines and chemokines. Among these, IL-1β and TNFα are important regulators of the acute sickness response, and consistent with the behavioral response observed in Supplemental Figure S1, plasma cytokines and mRNA transcripts of IL-1β and TNFα were increased in both Val66Val and Val66Met mice compared to controls at 2 h following LPS challenge. However, behavioral differences apparent in Val66Met mice at 24 h were paralleled by a significantly higher expression of inflammatory gene transcripts in the brain in Val66Met mice (Figure 2). These data suggest that the resolution of inflammation was impaired in Val66Met mice and could contribute to anhedonia-like behavior. Of note, no genotype × LPS interaction was apparent in peripheral cytokine protein levels, which suggests the Val66Met SNP does not impact peripheral immune regulation, but rather dysregulates neuroinflammation. These are the first data to reveal the specific effect of the BDNF Val66Met SNP on neuroinflammation. The results of this study build on our previous investigations, where we have reported that BDNF heterozygous mice have increased vulnerability to develop stress-induced anhedonia [18] and genetic reduction in BDNF was associated with reduced sucrose preference and increased neuroimmune response to peripheral immune challenge [19]. Importantly, inflammation alone does not explain the full extent of depressive symptoms [87], and not all individuals develop depressive symptoms even in the face of increased inflammation. It is plausible that a better understanding of the interaction between inflammation and genetic/environmental factors documented in this study will help resolve these gaps in knowledge.

This study has several strengths and limitations. We found that BDNF Val66Met mice exhibit an anhedonia-like behavioral response after LPS, while Val66Val mice did not (Figure 1). We ruled out the possibility of sickness behavior as a confounding factor, as evidenced by data in Supplemental Figure S1. Acute and extended sickness-related behaviors did not change across genotypes, suggesting that the trajectory of sickness did not affect behavioral testing. No differences in exploration capacity, body weight, or locomotion related to Val66Met SNP were observed, indicating the absence of a general lethargy effect that confounded behavioral testing. However, a more detailed time-course study warrants future studies to investigate this further. We did not find behavioral differences in the FST, but other studies have found increased immobility using this measure in Val66Met mice under stressed conditions [47,49]. However, the internal validity of FST in measuring depressive-like behaviors in rodent models is not robust, with variable results reported in the literature, and the FST has better sensitivity in assessing the effectiveness of antidepressant and anti-anxiety medications [88–90]. LPS-induced immune activation increased thigmotactic (wall-hugging) behavior in both genotypes. However, Val66Val mice were resilient to LPS challenge and did not exhibit anhedonia-like behavior (Figure 2A). The study by Notaras and colleagues found the Val66Val genotype to be resilient against mood maladaptation and required chronic corticosteroid administration to induce susceptibility to depressive behaviors [49]. Chen and colleagues observed an anxiety-like behavioral phenotype in BDNF Val66Met mice in the elevated plus maze; perhaps a stressor stronger than that of LPS like chronic corticosterone treatment is required to induce an anxiogenic phenotype [25,49]. Interestingly, humans expressing BDNF Val66Met show no baseline differences in several metrics of anxiety when compared to Val66Val subjects [91], and anxiety scores measured following pro-inflammatory treatment are comparable between Val66Val- and Val66Met-expressing individuals [30].

Previous studies have noted significant sex-based differences in the behavioral and transcriptomic elements between male and female humanized BDNF knock-in mice [92]. Of note, female BDNF Val66Met mice exhibit an anxiogenic phenotype at baseline in an age- and estrus cycle-dependent manner [93]. The results presented here were performed in young adult male humanized BDNF knock-in mice and limited the ability to comment on sex-based differences during an ongoing immune challenge. Regardless of limitations, the behavioral testing performed in male BDNF Val66Met mice did not reveal baseline performance deficits in sucrose preference or FST (Figure 2A,B). Future studies are required to investigate sex-based differences in neuroinflammation outcomes related to the BDNF Val66Met SNP. Specifically, future research should include testing of female mice at different ages and ovariectomized (with and without) female Val66Val and Val66Met mice under immune challenge conditions to address sex-based differences. The observed differences in behavioral and neuroinflammatory response reported in this study suggest that LPS-induced anhedonia-like behavior in Val66Met mice is only partially explained by localized changes in inflammatory gene transcripts measured in the hippocampus and nucleus accumbens (Figures 4 and 5). Interestingly, CD68 mRNA expression was remarkably different and suggests a role of microglia and/or infiltrating monocytes/macrophages in the dysregulated inflammatory process, but additional studies are needed to confirm this. Notably, inflammatory markers studied in whole-brain tissue (Figure 3) clearly indicate an upregulated expression in Val66Met mice treated with LPS. Speculatively, other brain regions such as the prefrontal cortex and the amygdala may be involved in mediating the neuroinflammatory response post-LPS challenge. While prior studies have implied compromised BBB integrity as a contributing factor in neuroinflammatory responses to peripheral immune challenge, our unpublished data in the C57B6/J background strain did not identify any BBB breakdown at the dose of LPS used in this study. If the Val66Met SNP renders the BBB more vulnerable to LPS-induced breakdown, it would likely impact the BBB throughout the brain, but these important questions remain to be directly investigated.

BDNF facilitates neuronal survival, differentiation, and proliferation and plays an important role in neurogenesis and plasticity [94]. The Val66Met SNP is the most extensively studied BDNF SNP, and it may be a genetic risk factor contributing to depression by interacting with stress or inflammation. Building on previous reports where BDNF-deficient mice exhibit protracted neuroinflammatory and behavioral responses following peripheral immune challenge with LPS [19,20], here, we provide evidence that BDNF Val66Met male mice exhibit prolonged anhedonia-like behavioral effects after LPS challenge, providing a good model to dissect the mechanism(s) of differential depression vulnerability. Overall, this study revealed a distinct SNP × LPS interaction that prolonged elements of the neuroinflammatory response, while no differences were observed in the peripheral inflammatory response or the acute sickness-related behavioral response. In conclusion, these data support that the BDNF Val66Met polymorphism may be an important contributor to increased vulnerability in the development of inflammation-associated anhedonia-like behavior. The sustained elevation of IL-1β and TNFα expression, specifically in the brain, without significant differences in peripheral cytokine responses, suggests that genetic vulnerability factors such as Val66Met may impair the resolution of neuroinflammation independently of peripheral inflammatory resolution. This may be particularly relevant when considering the potential implications of chronic or cumulative exposure to poorly resolved/regulated neuroinflammatory responses across the lifespan. These findings could have implications for individuals with the Val66Met polymorphism who might benefit from therapeutic strategies targeting central (rather than solely peripheral) inflammatory processes. Our results advocate for additional studies that investigate personalized treatment approaches in depression, where inflammatory biomarkers and genetic screening for BDNF polymorphisms can inform the use of anti-inflammatory or neurotrophic-based interventions (e.g., TNF antagonists, IL-1 blockers, or agents promoting BDNF signaling such as TrkB agonists).

## Supplementary Material

1

## Figures and Tables

**Table 1.** Peripheral immune response is not influenced by BDNF Val66Met polymorphism genotypes. Plasma concentrations of the pro-inflammatory cytokines IL-1β, IL-6, and TNFα at 2 h (n = 3–5 mice/group) and 24 h (n = 6–15 mice/group) after saline or LPS administration, and data were analyzed using a two-way ANOVA with genotype and LPS as independent factors.

	Val/Val		Val/Met		Main Effects		Effect Size
	Saline	LPS	Saline	LPS			
	Mean (SEM)	Mean (SEM)	Mean (SEM)	Mean (SEM)	Genotype *p*-Value	Treatment *p*-Value	η^2^p
**2 h**							
IL-1β	0.152 (0.152)	7.596 (6.078)	0.543 (0.543)	4.381 (4.381)	0.2413	0.7630	na
IL-6	1.379 (0.939)	195.172 (0.0)	1.500 (0.815)	152.397 (42.776)	0.3461	<0.0001 ****	0.839
TNFα	0.783 (0.783)	73.288 (19.506)	11.628 (11.533)	40.409 (23.830)	0.5309	0.0111 *	0.402
**24 h**							
IL-1β	2.841 (0.998)	2.315 (1.037)	3.460 (1.042)	4.678 (1.903)	0.8070	0.2981	na
IL-6	0.539 (0.173)	72.034 (27.041)	0.956 (0.620)	75.048 (17.211)	0.9191	0.0001 ***	0.350
TNFα	1.296 (0.746)	3.292 (1.370)	0.543 (0.543)	4.211 (0.644)	0.9292	0.0064 **	0.345

Na—not applicable * p < 0.05, ** p < 0.01, *** p < 0.001, **** p < 0.0001.

## Data Availability

Individual data points are provided in the figures, and raw data used to generate the summary findings of this study are available from the corresponding author—Jason C. O’Connor, PhD, oconnorj@uthscsa.edu—upon request.

## Main Points:

- The Val66Met genotype did not affect peripheral inflammatory response, acute neuroinflammation, or acute sickness behavior response to lipopolysaccharide.
- The Val66Met genotype exacerbates LPS-induced anhedonia-like behavior and the neuroinflammatory response.
- BDNF Val66Met polymorphism may disrupt negative regulation of inflammation following peripheral immune challenge.
