# Supplementary material for: Dysregulated Neuroimmune and Anhedonia-Like Behavioral Response Following Peripheral Immune Challenge in Mice Carrying the Val66Met Brain-Derived Neurotrophic Factor Polymorphism
Source: Psychiatry Int (Basel). Author manuscript; Available in PMC 2026 Aug 8. (PMC13450145; doi:10.3390/psychiatryint6030087)
Supplement: Supplementary file 1 [file NIHMS2189357-supplement-1.pdf]

Supplementary Materials: The following supporting information can be downloaded at https://www.mdpi.com/article/doi/s1, Figure S1: Acute sickness behavior was unchanged among BDNF genotypes with LPS challenge; Table S1: Other mRNA transcripts measured in whole brain.

Supplemental Table 1. Other mRNA transcripts measured in whole brain. 24 hours after LPS challenge, whole brain mRNA was analyzed using RT-qPCR for the following targets and data were analyzed using a two-way ANOVA with genotype and LPS as independent factors - Inducible nitric oxide synthase (iNOS), CD68, interleukin-6 (IL-6), interleukin-1 receptor antagonist (IL-1Rn), interleukin receptor-4 alpha (IL-4Rα). * indicates $p<0.05$.

	Val/Val		Val/Met		Main Effects	
	Mean (SD)	Mean (SD)	Mean (SD)	Mean (SD)	Genotype	Treatment
Target	Saline	LPS	Saline	LPS	*p*-value	*p*-value
iNOS	1.2 (0.6)	1.2 (0.3)	1.3 (0.5)	1.2 (0.2)	0.1718	0.4379
CD68	1.2 (0.7)	3.5 (0.6)	1.6 (0.1)	4.4 (0.8)	0.2796	0.1198
IL-6	1.0 (0.2)	0.7 (0.4)	1.4 (0.9)	0.4 (0.1)	0.5086	0.4304
IL-1Rn	1.0 (0.5)	23.1 (7.7)	2.5 (1.5)	22.8 (1.5)	0.8347	<0.0001*
IL-4Rα	1.0 (0.4)	2.3 (0.5)	1.1 (0.4)	2.7 (0.6)	0.5145	0.0001*

Supplemental Figure 1. Acute sickness behavior was unchanged among BDNF genotypes with LPS challenge. Behavioral testing was conducted 2 hours following saline or LPS administration, and data were analyzed using a two-way ANOVA with genotype and LPS as independent factors for normally distributed data and non-parametric Kruskal-Wallis's test were used to analyze non-normally distributed data. (A) Locomotion in the open field, main effects of LPS: (1,20) = 33.81, $p<0.0001$ and (B) social exploration, Kruskal-Wallis's test $H(3) = 10.24$, $p=0.0166$. n =5-7 mice/group. Post-hoc testing with Tukey's multiple comparison test to compare differences between group means if main effects of genotype or LPS were detected and for non-normally distributed data post-hoc testing with corrected Dunn's multiple comparison test was used to compare differences between group means; # represents main effect of LPS; n.s. - not significant; *$p<0.05$, ***$p<0.001$.

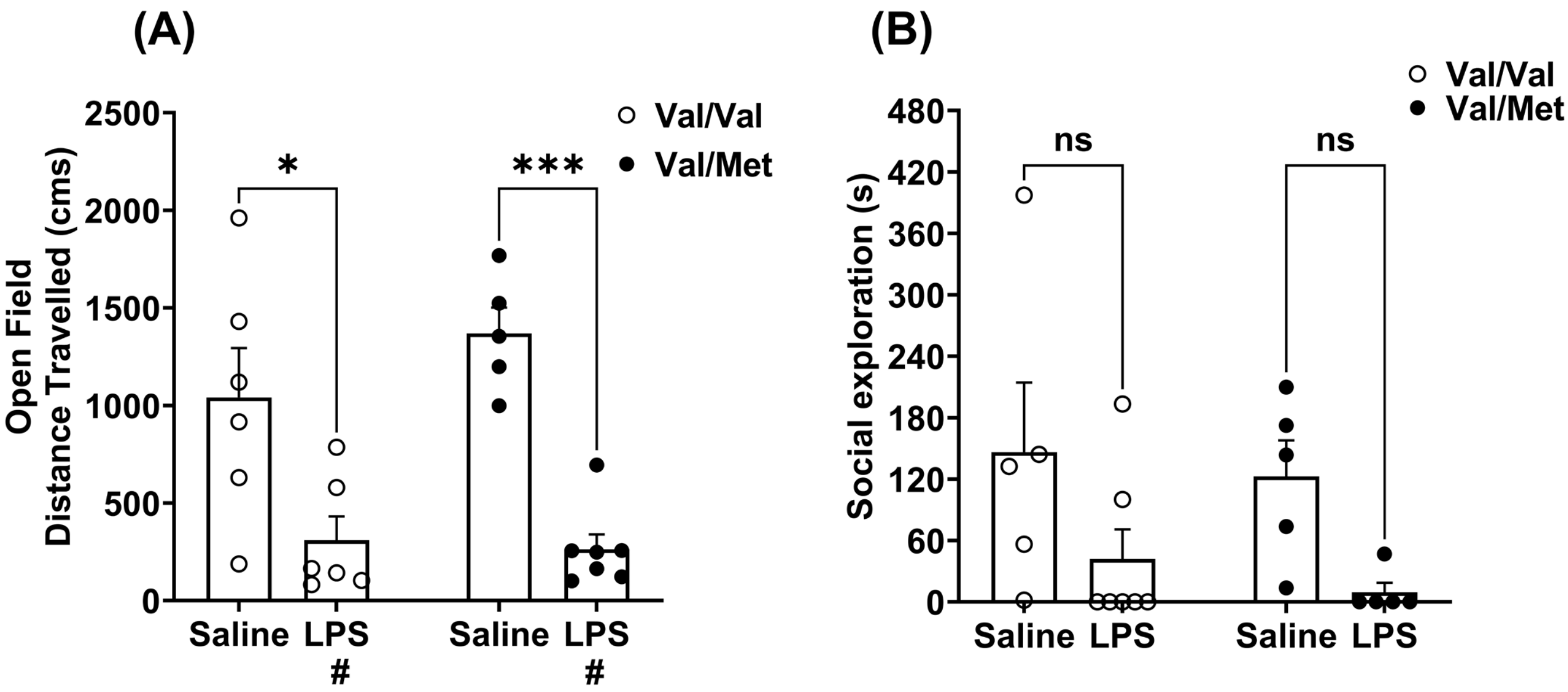
(A)
Val/Val
Val/Met
Open Field
Distance Travelled (cms)
2500
2000
1500
1000
500
0
*
***
Saline
LPS
#
Saline
LPS
#
(B)
Val/Val
Val/Met
Social exploration (s)
480
420
360
300
240
180
120
60
0
ns
ns
Saline
LPS
Saline
LPS
